# Reversible Lesions of the Genu of the Corpus Callosum and White Matter Detected by MRI in a Neonate With Apnea: A Case Report

**DOI:** 10.7759/cureus.107872

**Published:** 2026-04-28

**Authors:** Toi Ono, Seigo Korematsu, Mai Sekine, Keiko Mizuta, Yasuko Urushihara, Satoshi Masutani, Yuta Uchida, Shingo Kobayashi, Kohei Osada, Yoshio Sakurai, Wataru Watanabe

**Affiliations:** 1 Department of Pediatrics, Saitama Medical Center, Saitama Medical University, Kawagoe, JPN; 2 Department of Pediatric Intensive Care, Saitama Medical Center, Saitama Medical University, Kawagoe, JPN; 3 Department of Radiology, Saitama Medical Center, Saitama Medical University, Kawagoe, JPN

**Keywords:** apnea, corpus callosum lesion, mild encephalitis/encephalopathy with a reversible splenial lesion (mers), reversible splenial lesion syndrome, term neonate

## Abstract

In this report, we describe a 19-day-old female neonate who presented with recurrent apnea, poor feeding, and cyanosis. Brain diffusion-weighted MRI revealed hyperintense lesions in the genu of the corpus callosum and bilateral frontal white matter with corresponding low apparent diffusion coefficient values. Antibiotic therapy and supportive management were initiated, and the apnea resolved by hospital day 6. A follow-up brain MRI performed on day 49 demonstrated the complete resolution of the previously observed lesions. No neurological sequelae were observed up to 11 months of age.

This case illustrates a rare presentation of neonatal-onset apnea associated with reversible lesions localized in the genu of the corpus callosum on MRI. Several differential diagnoses were considered, including mild encephalitis/encephalopathy with a reversible splenial lesion (MERS)-related conditions, viral encephalitis, postictal changes, and pre-Wallerian degeneration. When reversible corpus callosum lesions are observed in neonates, a variety of underlying pathophysiological processes should be considered in the differential diagnosis.

## Introduction

Reversible lesions of the corpus callosum identified on MRI have been described using several terms, including mild encephalitis/encephalopathy with a reversible splenial lesion (MERS), reversible splenial lesion syndrome (RESLES), and cytotoxic lesions of the corpus callosum (CLOCC) [1-3]. These entities are associated with diverse underlying conditions, such as infectious or autoimmune encephalitis/encephalopathy, seizures, medications, and metabolic disturbances. In most cases, patients exhibit a benign clinical course and favorable outcomes. However, because the clinical manifestations and MRI findings often overlap, distinguishing between these conditions can be challenging [3,4].

In this report, we describe the case of a neonate who presented with recurrent apnea and reversible lesions involving the genu of the corpus callosum and bilateral frontal white matter on diffusion-weighted imaging.

## Case presentation

A female neonate was delivered at 39 weeks and six days of gestation by scheduled cesarean section due to a previous cesarean delivery (birth weight, 3,082 g; birth length, 49.3 cm). The prenatal and family histories were unremarkable. She received oral vitamin K₂ weekly.

At 19 days of age, the patient suddenly developed poor feeding and perioral cyanosis and was poorly responsive to stimulation. No upper respiratory symptoms, such as rhinorrhea or cough, were observed before admission. After initial evaluation in the outpatient clinic, she was referred to our hospital. Owing to recurrent apnea, she was admitted to the pediatric intensive care unit (PICU) for further evaluation and management.

On admission, her vital signs were as follows: body temperature, 36.8°C; heart rate, 150 beats/minute; blood pressure, 56/30 mmHg; oxygen saturation, 99% on room air; and respiratory rate, 40-50 breaths/minute. She developed frequent apnea without respiratory effort, accompanied by oxygen desaturation to 65% and bradycardia with a heart rate of 90 beats/minute. Each episode resolved within 1 minute with tactile stimulation but recurred every few minutes.

A physical examination revealed no remarkable findings, with a flat anterior fontanelle, clear lung fields, no cardiac murmurs, a soft abdomen, and warm extremities. Neurological examination revealed normal muscle tone, symmetric spontaneous movements, and intact primitive reflexes, with no focal neurological deficits. No abnormal eye movements or asymmetry were observed. Mildly decreased activity was noted between apnea episodes.　

Laboratory findings on admission (Table 1) revealed a white blood cell count of 7,400/µL, a hemoglobin level of 13.0 g/dL, and a platelet count of 285 × 10³/µL. Liver enzyme levels were within normal limits, whereas the C-reactive protein level was mildly elevated at 0.53 mg/dL. A serum electrolyte analysis revealed hyponatremia at 132 mEq/L, whereas potassium and chloride levels were within normal ranges at 4.9 and 98 mEq/L, respectively. Hypoglycemia and hyperammonemia were not observed. Venous blood gas findings were consistent with respiratory acidosis, with a pH of 7.308, pCO_2_ of 62.1 mmHg, and HCO_3_⁻ level of 31.1 mmol/L.

**Table 1:** Blood test. Blood gas analysis was performed using a venous sample.

Test	Value	Reference range	Unit
Total protein	5.3	4.7-5.9	g/dL
Albumin	3.6	3.0-4.1	g/dL
Aspartate aminotransferase	21	20-71	U/L
Alanine aminotransferase	13	11-68	U/L
Lactate dehydrogenase	214	314-737	U/L
Creatinine	0.18	0.20-0.40	mg/dL
Blood urea nitrogen	2.1	9.0-19.0	mg/dL
Sodium	132	135-150	mEq/L
Chloride	98	98-109	mEq/L
Potassium	4.9	4.3-5.5	mEq/L
Glucose	105	80-100	mg/dL
C-reactive protein	0.53	<0.20	mg/dL
Ammonia	84	30-80	mg/dL
Lactate	10	3.0-17.0	mg/dL
Pyruvate	0.9	0.3-0.9	mg/dL
White blood cell	7.4	6.0-19.0	×10^3^/μL
Hemoglobin	13.0	13.0-15.0	g/dL
Platelet	285	150-400	×10^3^/μL
Activated partial thromboplastin time	40.3	30-45	seconds
Prothrombin time	9.7	11-13	seconds
Fibrinogen	274	95-245	mg/dL
Fibrin degradation products	2.00	<5.00	μg/mL
D-dimer	0.40	<1.00	μg/mL
pH	7.308	7.35-7.45	
pCO_2_	62.1	35-45	mmHg
HCO_3_^-^	31.1	23-29	mmol/L
BE	3.1	-2.0-3.0	mmol/L

**Figure 1:**
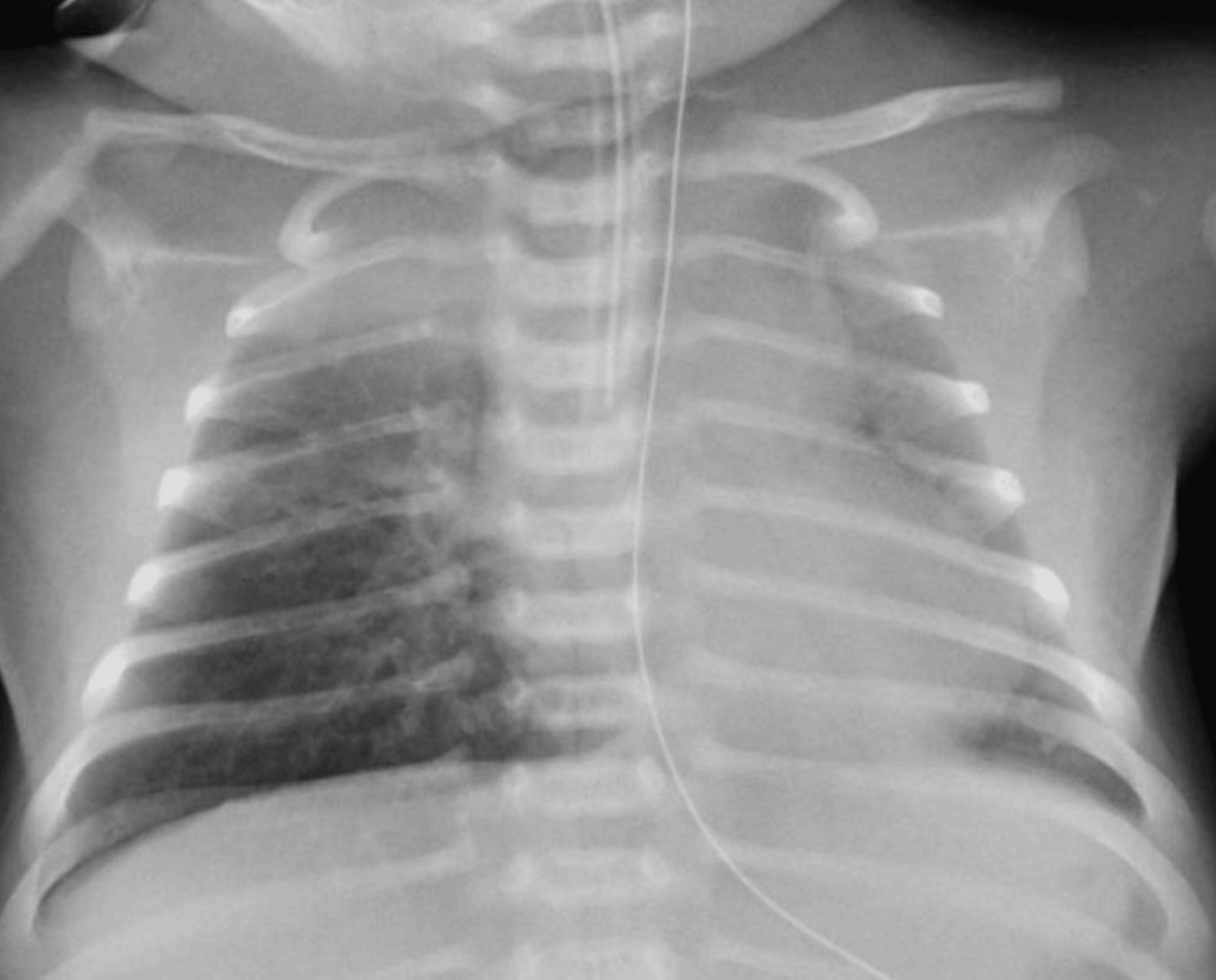
Chest radiograph on hospital day 2 showing infiltrative shadows in the right upper lung field and hilar region.

A cerebrospinal fluid (CSF) analysis (Table 2) showed no pleocytosis, protein elevation, or low glucose levels, and the FilmArray® (BioFire Diagnostics, LLC, Salt Lake City, UT) meningitis panel (Table 3) detected no pathogens. Chest radiography performed on hospital day 1 showed no abnormalities; however, infiltrative shadows appeared in the right upper lung field and hilar region on hospital day 2 (Figure 1). A FilmArray® respiratory panel test of a nasopharyngeal swab (Table 3) was positive for human rhinovirus (HRV)/enterovirus (EV). Blood, sputum, and CSF cultures were negative for bacterial growth. Transthoracic echocardiography showed no congenital heart disease, and the systolic function was preserved. Non-contrast brain computed tomography showed no abnormalities, including cerebral edema.

**Table 2:** Cerebrospinal fluid analysis.

Parameter	Result	Reference range	Unit
Appearance	Clear		
Cell count	1	0-22	/μL
Mononuclear cells	100		%
Polymorphonuclear cells	0		%
Protein	69	55-158	mg/dL
Glucose	89	31-70	mg/dL

**Table 3:** FilmArray® (BioFire Diagnostics, LLC, Salt Lake City, UT) results.

Parameter	Result
FilmArray® Respiratory Panel	Positive for human rhinovirus/enterovirus only
FilmArray® Meningitis Panel	All negative

**Figure 2:**
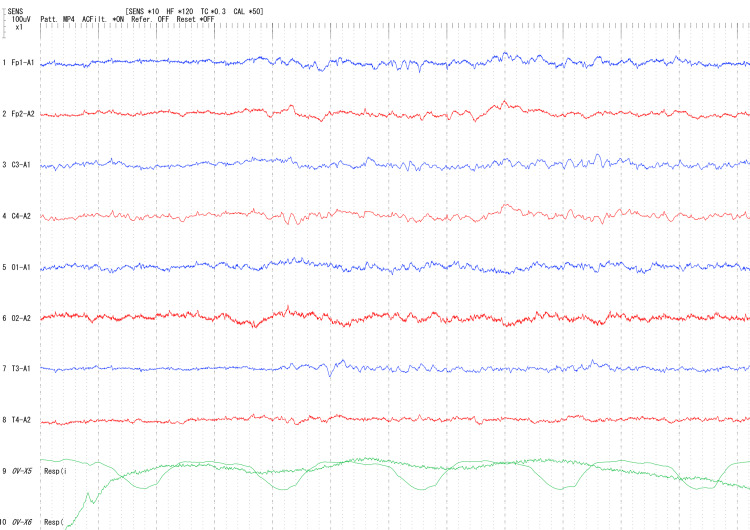
Continuous electroencephalography (EEG) during midazolam sedation showing low-voltage background activity and poorly developed sleep-wake cycling, with no epileptiform discharges and no apnea episodes during monitoring.

Continuous electroencephalography (EEG) during midazolam sedation (Figure 2) showed low-voltage background activity and poorly developed sleep-wake cycling; no epileptiform discharges were observed. EEG was not repeated after midazolam was discontinued. No apnea episodes occurred during EEG monitoring.

**Figure 3:**
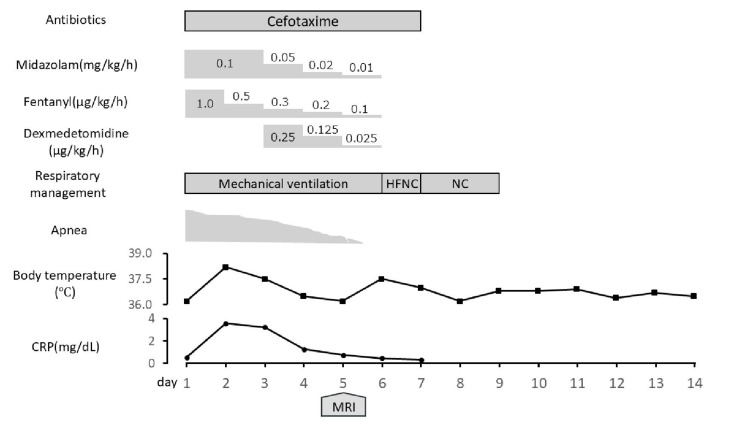
Clinical course of treatment, symptoms, and laboratory findings. This figure was created using Microsoft PowerPoint. CRP, C-reactive protein; NC, nasal cannula; HFNC, high-flow nasal cannula

**Figure 4:**
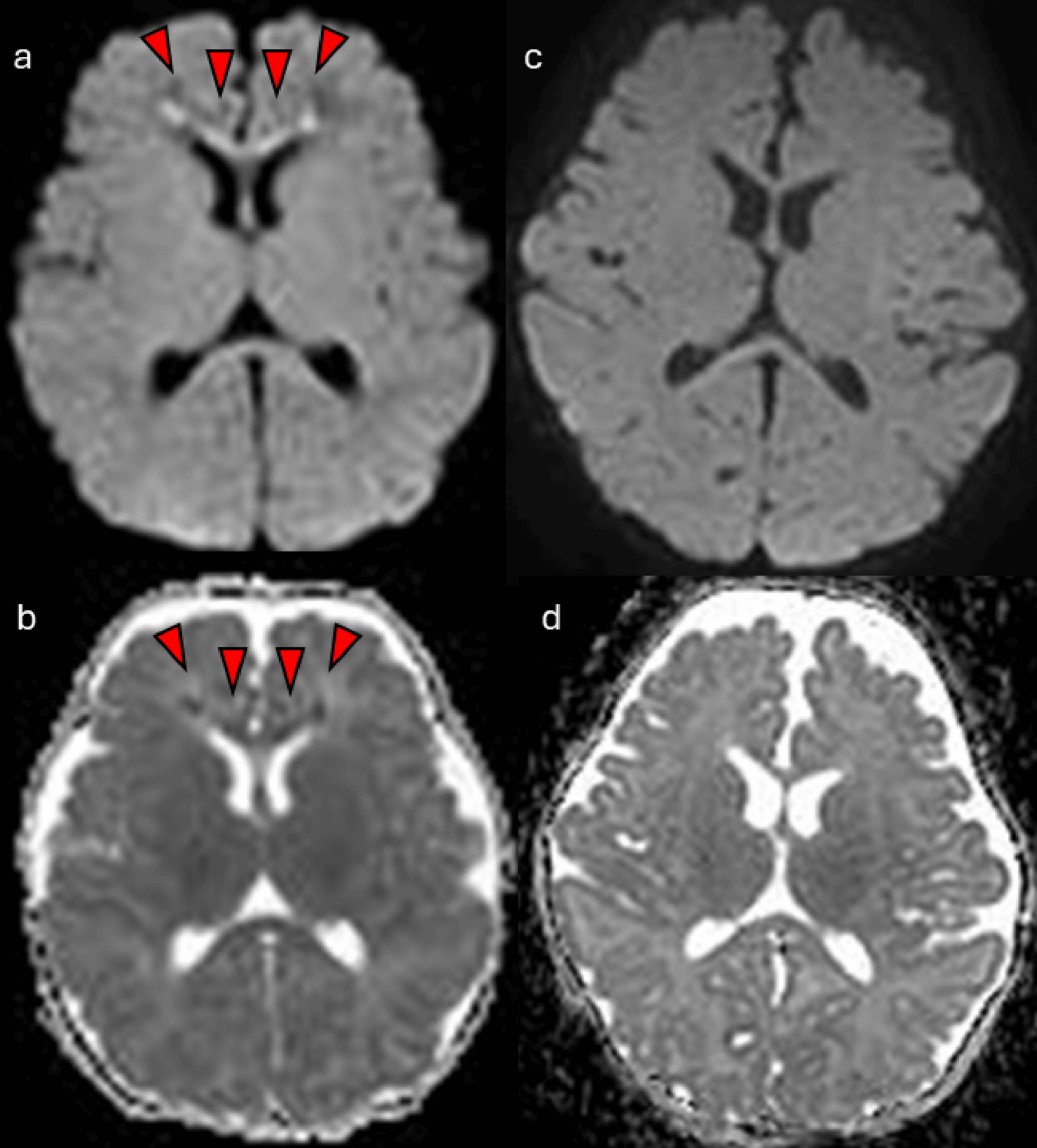
Serial brain MRI findings. Upper row: Diffusion-weighted imaging (DWI). Lower row: Apparent diffusion coefficient (ADC) maps. (a, b) Day 5 after onset: Hyperintense signals (red arrows) are observed in the genu of the corpus callosum and bilateral frontal white matter on DWI, with corresponding low ADC values (red arrows). (c, d) Day 49 after onset: The abnormal signals in the genu of the corpus callosum and bilateral frontal white matter have resolved.

After admission (Figure 3), mechanical ventilation was initiated for apnea, and continuous sedation with midazolam was administered. Cefotaxime was administered at a dose of 150 mg/kg/day for pneumonia. To evaluate ongoing apnea, ventilator settings were adjusted daily, with transitions to spontaneous breathing modes as appropriate. As apnea persisted until hospital day 5, a brain MRI was performed for further evaluation (Figure 4).

Brain MRI revealed no intracranial mass lesions or abnormalities in the brainstem. However, diffusion-weighted imaging revealed hyperintense lesions in the genu of the corpus callosum and bilateral frontal white matter, accompanied by corresponding low apparent diffusion coefficient (ADC) values. Apnea resolved on hospital day 6, and no additional treatments, including corticosteroids, were initiated. The patient was extubated on the same day. Subsequently, she remained free of apnea and altered mental status and was discharged on hospital day 14.

Follow-up brain MRI performed on day 49 showed complete resolution of the previously observed lesions in the genu of the corpus callosum and bilateral frontal white matter (Figure 4). At 11 months of age, she exhibited normal development.

## Discussion

This neonate presented with apnea, and a nasopharyngeal swab tested positive for HRV/EV. Apnea episodes persisted even during mechanical ventilation, suggesting a central origin rather than an obstructive cause. The EEG showed abnormalities in background activity, and the brain MRI demonstrated transient lesions in the genu of the corpus callosum and white matter.

Several differential diagnoses were considered, including MERS-related conditions, viral encephalitis, autoimmune encephalitis, peri-ictal MRI changes associated with seizures, and pre-Wallerian degeneration. The differential diagnoses and their distinguishing features are summarized in Table 4.

**Table 4:** Characteristics and distinguishing features of the differential diagnoses in the present case. Sources: [1,5,13,18,19,21,22,27,28]. MERS, mild encephalitis/encephalopathy with a reversible splenial lesion; GFAP, glial fibrillary acidic protein; FLAIR, fluid-attenuated inversion recovery

Differential diagnosis	Pathophysiology	Brain MRI findings
MERS-related disorders	Transient intramyelinic or axonal edema triggered by preceding infection or other insults	Transient hyperintensity in the splenium of the corpus callosum on diffusion-weighted imaging
Viral encephalitis	Inflammatory parenchymal brain injury caused by viral infection	Hyperintense lesions on T2-weighted/FLAIR images and diffusion-weighted imaging
Autoimmune encephalitis	Encephalitis/encephalopathy caused by autoimmune mechanisms directed against the central nervous system	Hyperintense lesions on T2-weighted/FLAIR images. In anti-GFAP antibody–positive cases, transient hyperintensity in the corpus callosum or periventricular white matter on diffusion-weighted imaging
Postictal MRI changes	Transient cytotoxic or vasogenic edema in regions associated with seizure activity	Transient hyperintensity on diffusion-weighted imaging at the seizure focus or along the propagation pathways
Pre-Wallerian degeneration	Secondary axonal and myelin degeneration following primary brain injury	Hyperintensity on diffusion-weighted imaging in neural tracts distal to the primary lesion. In mild cases, the lesions may be transient
Present case	Central apnea triggered by rhinovirus/enterovirus infection	Transient hyperintensity on diffusion-weighted imaging in the genu of the corpus callosum and bilateral frontal white matter

The relationship between the patient’s apnea and the MRI findings remains unclear. Although the apnea episodes are suggestive of a central origin, a possible association with these differential diagnoses that may cause such MRI findings, as well as infectious etiologies, should be considered.

MERS typically develops in childhood after antecedent infection and is characterized by reversible hyperintensity in the splenium of the corpus callosum on diffusion-weighted imaging. The proposed mechanisms underlying MERS include intramyelinic edema and axonal swelling [1-5]. However, patients who follow a clinical course similar to that of MERS but show no splenial involvement and instead demonstrate reversible hyperintense lesions confined to the genu of the corpus callosum are classified as having MERS-related conditions.

Two similar cases have been reported. Yağmur et al. described a 34-year-old woman who presented with seizures and impaired consciousness and showed a transient lesion confined to the genu of the corpus callosum on diffusion-weighted imaging [6]. Sasaki et al. reported an eight-year-old boy who presented with abnormal behavior and impaired consciousness and demonstrated a transient lesion limited to the genu on diffusion-weighted imaging [7]. Similar to MERS, these lesions were probably caused by intramyelinic edema and axonal swelling. The genu, like the splenium, may be particularly susceptible to edematous changes because it contains smaller-diameter axons and has a higher packing density than the body of the corpus callosum [8].

Although MERS with neonatal onset is rare, five neonatal cases have been reported to date [9]. In addition, one previous case of MERS presented with apnea similar to the present case. Li et al. described a one-year-old boy who developed seizures and apnea triggered by respiratory syncytial virus infection and was diagnosed with MERS based on brain MRI findings. Because airway obstruction was absent and ocular deviation occurred during seizure and apnea episodes in association with EEG abnormalities, apnea was considered to be of central origin [10]. These reports suggest that MERS-related conditions may explain the present case.

Viral encephalitis represents another important differential diagnosis. Neonatal viral infections typically cause nonspecific symptoms, such as fever, lethargy, or poor feeding, and some cases progress to severe sepsis-like illness [11,12]. Central nervous system manifestations, including seizures, may occur, and abnormalities may be detected on a CSF analysis, brain MRI, and EEG [13]. However, in neonates and young infants, particularly those with EV or human parechovirus (HPeV) infection, CSF pleocytosis is frequently absent. Younger age and earlier timing after symptom onset increase the likelihood of a normal CSF cell count [14-16]. This phenomenon has been attributed to the immaturity of neonatal immune responses and reduced leukocyte migration into the CSF [15]. EV- and HPeV-associated encephalitis often causes reversible hyperintensity in the periventricular white matter and corpus callosum on diffusion-weighted imaging, complicating differentiation from MERS [17]. In this case, the CSF analysis showed neither pleocytosis nor protein elevation; however, the positive HRV/EV result on the nasopharyngeal swab raised the possibility of EV-associated viral encephalitis. However, EV was not detected in the CSF via the FilmArray® meningitis/encephalitis panel. Previous studies have demonstrated the high sensitivity and specificity of this assay for EV. Therefore, clear evidence supporting viral encephalitis was not obtained [16].

Autoimmune encephalitis is another important differential diagnosis. Autoimmune encephalitis typically presents with a subacute onset of diverse neurological symptoms, including impaired consciousness and seizures, mediated by autoantibodies or immune responses directed against the central nervous system. Diagnostic findings may include the detection of specific autoantibodies, abnormalities on brain MRI, CSF abnormalities, and EEG abnormalities [18]. In particular, patients positive for anti-glial fibrillary acidic protein (GFAP) antibodies may show reversible hyperintense lesions in the periventricular white matter and corpus callosum on diffusion-weighted imaging, which can resemble the imaging features of MERS [19]. However, autoimmune encephalitis during the neonatal period is extremely rare, and reported neonatal cases have involved the transplacental transfer of maternal autoantibodies [20]. In this case, the mother had no clinical manifestations suggestive of autoimmune disease; therefore, autoantibodies, including anti-GFAP antibodies, were not evaluated.

Peri-ictal MRI findings following seizures represent another important differential diagnosis. These changes manifest as transient hyperintense signals on diffusion-weighted imaging in seizure-related cortical regions and usually resolve spontaneously within several days to weeks [21-22]. The corpus callosum, which serves as the major commissural fiber tract connecting the bilateral cerebral hemispheres, may show transient MRI abnormalities even when it is not the primary seizure focus, presumably because of seizure propagation [23]. Previous studies have reported transient diffusion abnormalities localized to the body or genu of the corpus callosum in neonates and infants [24,25]. Kubota et al. described a neonate on day 2 of life without a history of birth asphyxia who developed transient hyperintense lesions in the genu and splenium of the corpus callosum on diffusion-weighted imaging after a single seizure [24]. Okumura et al. reported two infants with benign partial epilepsy in infancy who showed transient hyperintense lesions in the genu and splenium after seizure clusters [25]. Nguyen et al. analyzed 123 neonates with hypoxic-ischemic encephalopathy and demonstrated a strong association between seizures and transient diffusion restriction in the splenium of the corpus callosum [26]. These findings suggest that MRI changes in the corpus callosum may reflect seizure activity rather than the severity of the underlying disease. Collectively, these reports indicate that transient corpus callosum lesions can occur after seizures in neonates regardless of the underlying etiology. In this case, although the EEG showed no epileptiform discharges, background EEG abnormalities were present, and seizure activity cannot be completely excluded. However, because EEG was not repeated after discontinuation of midazolam, the potential influence of sedation on EEG findings cannot be completely excluded.

Pre-Wallerian degeneration represents the final differential diagnosis. Wallerian degeneration refers to the secondary degeneration of distal axons and myelin following primary cortical injury, which ultimately leads to chronic atrophy. Pre-Wallerian degeneration represents an early stage of this process and appears as hyperintense lesions on diffusion-weighted imaging within days of onset. In neonates, pre-Wallerian degeneration has been reported as an early imaging marker and prognostic indicator after cortical injury, such as hypoxic-ischemic encephalopathy [27,28]. Although cases that progress to established Wallerian degeneration often result in irreversible neurological deficits, mild cases may show resolution of MRI abnormalities within one to two weeks without neurological sequelae [29-31]. Lesions involving the corticospinal tracts are strongly correlated with neurological impairment, whereas corpus callosum lesions occur more frequently in mild cases and in patients with favorable outcomes [30,31]. In this case, no definitive primary cortical lesion was identified; however, mild pre-Wallerian degeneration cannot be excluded as a possible contributor to the observed MRI findings.

## Conclusions

This neonate presented with reversible lesions involving the genu of the corpus callosum and white matter, as detected on MRI with recurrent apnea. In this case, several differential diagnoses were considered, including MERS-related conditions, viral encephalitis, peri-ictal changes, and pre-Wallerian degeneration. When reversible corpus callosum lesions are observed in neonates, the differential diagnosis is broad, and overlapping pathophysiological processes may make accurate diagnosis challenging.
